# Multi-Level Immune Support by Vitamins C and D during the SARS-CoV-2 Pandemic

**DOI:** 10.3390/nu14030689

**Published:** 2022-02-06

**Authors:** Anitra C. Carr, Adrian F. Gombart

**Affiliations:** 1 Nutrition in Medicine Research Group, Department of Pathology and Biomedical Science, University of Otago, Christchurch 8011, New Zealand; 2 Linus Pauling Institute, Oregon State University, Corvallis, OR 97331, USA; adrian.gombart@oregonstate.edu; 3 Department of Biochemistry and Biophysics, Oregon State University, Corvallis, OR 97331, USA

**Keywords:** SARS-CoV-2, COVID-19, vitamin C, vitamin D, pneumonia, sepsis, long COVID, immunisation, immune support

## Abstract

Vitamins C and D have well-known immune supportive roles, with deficiencies in both vitamins predisposing to increased risk and severity of respiratory infections. Numerous studies have indicated that administration of these vitamins, particularly to people who are deficient, can decrease the risk and severity of respiratory infections. This has stimulated an interest in the potential efficacy of these vitamins in people with novel coronavirus (SARS-CoV-2) infection and its more severe disease (COVID-19). In this overview, we highlight the current research evidence around the multiple levels of immune support provided by vitamins C and D in the context of general respiratory infections and with a focus on the current SARS-CoV-2 pandemic. These include: prevention of infection; attenuating infection symptoms and severity; adjunctive therapy for severe disease; attenuating ongoing sequelae (long COVID); and immunisation support. Although some of these topics have not yet been investigated in great depth concerning SARS-CoV-2 and COVID-19, extensive research into the role of these vitamins in general respiratory infections has highlighted directions for future research in the current pandemic.

## 1. Introduction

Since the novel coronavirus (SARS-CoV-2) and the associated disease (COVID-19) were declared a global pandemic in early 2020, there has been a worldwide effort to establish therapies to prevent and treat the respiratory infection and more severe disease. Although vaccination status is now high in most high-income countries, vaccination rates are still relatively low in many low-middle income countries. Furthermore, despite high vaccination rates in much of the developed world, SARS-CoV-2 infection cases and COVID-19 morbidity and mortality remain high. As such, additional means of supporting optimal immune function are crucial.

Both the innate and adaptive immune systems are absolutely reliant on appropriate nutritional support for optimal functioning [1]. Of the various immune-supportive micronutrients, vitamins C and D are two of the most well-established [2,3]. Deficiencies of these vitamins are common in many regions of the world and within specific subpopulations [4,5]. Deficiencies of in these vitamins are known to impair the immune system, resulting in severe respiratory infections [6,7,8]. In the case of vitamin C, pneumonia is a common complication of chronic vitamin C deficiency (scurvy) and is one of the most common causes of mortality in people with scurvy [6]. In addition, infection itself can cause further depletion of micronutrients, particularly vitamin C, thus necessitating higher intakes to restore optimal micronutrient status [9]. 

In this overview, we highlight the current evidence around the multiple levels of immune support provided by vitamins C and D in the context of general respiratory infections and with a focus on the current SARS-CoV-2 pandemic. These include: prevention of infection; attenuating infection symptoms and severity; adjunctive therapy for severe disease; attenuating ongoing sequelae (long COVID); and immunisation support. Although some of these topics have not yet been investigated in great depth concerning SARS-CoV-2 and COVID-19, extensive research into the role of these vitamins in general respiratory infections has highlighted directions for future research in the current pandemic. 

## 2. Prevention of Respiratory Infection

### 2.1. Vitamin C

Coronaviruses are one of the many types of viruses that can cause the common cold [10]. Meta-analysis of 24 trials investigating vitamin C for the prevention of the common cold has indicated that prophylactic supplementation with doses ≥200 mg/day did not decrease the incidence of the common cold in the general population [11]. In the case of SARS-CoV2 infection, one case-control study has attempted to estimate the effect of regular vitamin C supplementation on the incidence of SARS-CoV-2 infection [12]. Cases and controls were health-care workers who tested positive and negative, respectively, for SARS-CoV-2 infection. Of the 372 participants, 67 participants took vitamin C supplements (500 mg) once or twice daily. There was, however, no significant association with SARS-CoV-2 infection compared with the control group. 

In contrast, meta-analysis of a handful of trials in people under enhanced physical stress who were regularly supplementing with vitamin C indicated a >50% decrease in the incidence of colds [11]. This may be particularly relevant in cases of severe infection where vitamin C appears to act as part of the body’s stress response [13]. Psychological stress is also known to negatively affect the immune system [14], and could potentially enhance requirements for vitamin C [15]. Thus, future research around the role of vitamin C in the prevention of SARS-CoV-2 infection should specifically focus on subgroups who are under enhanced physical or psychological stress and who are at risk of vitamin C deficiency [16].

### 2.2. Vitamin D

It is clear that an inverse association exists between vitamin D status and the risk of acute respiratory tract infections [17]. Similarly, meta-analysis of 54 studies and 1,403,715 patients has indicated that those with low vitamin D levels had a higher susceptibility to SARS-CoV-2 infection and associated hospitalisation [18]. However, due to inherent study limitations caution should be exercised in interpreting the results as another meta-analysis with 11 cohort studies and 536,105 patients, did not show a link between vitamin D deficiency or insufficiency and susceptibility to SARS-CoV-2 infection [19]. 

It should be noted that meta-analyses of trials investigating vitamin D supplementation and risk of acute respiratory tract infection have indicated that those with a low vitamin D status at the start of the trial tend to achieve better results following supplementation [20,21]. Furthermore, recent meta-analyses show supplementation protects against acute respiratory infections when given as daily, but not as an intermittent bolus dose [21,22]. These aspects should be taken into consideration during the design of future SARS-CoV-2 trials.

## 3. Attenuating Infection Symptoms and Severity

### 3.1. Vitamin C

In the absence of specific comorbidities that increase the risk of developing severe COVID-19 [23], infection with SARS-CoV-2 can be relatively mild and even asymptomatic [24]. To date, one published trial has investigated the effects of vitamin C supplementation on SARS-CoV-2 symptoms [25]. This trial was underpowered as it was halted early, but nevertheless showed a non-significant 1.2-day decrease in duration to reach 50% reduction in symptoms in the participants who received supplemental vitamin C (8 g/day). Furthermore, independent statistical analysis of the results showed a significant 70% increase in the rate of recovery in the vitamin C group compared to standard care [26]. Similarly, in the case of the common cold, meta-analysis of 31 trials indicated that prophylactic vitamin C supplementation in the general population can decrease both the duration and severity of the common cold, with up to an 18% decreased duration in children who received ≥1 g/day [11]. Supplementation with gram doses of vitamin C following initiation of cold symptoms also provided a dose-dependent decrease in the duration of the common cold [11]. 

Pneumonia is a common complication of severe respiratory infections, including severe SARS-CoV-2 infection [27]. Epidemiological evidence has suggested that people in the highest quartile of vitamin C status had a lower incidence of pneumonia than those in the lowest quartile [28]. Furthermore, meta-analysis has indicated that prophylactic supplementation with vitamin C can decrease the risk of developing pneumonia [29]. As such, regular vitamin C supplementation may attenuate progression of mild SARS-CoV-2 infection to the more severe complication of pneumonia observed in COVID-19. 

### 3.2. Vitamin D

Meta-analysis of eight observational studies has suggested an association between vitamin D deficiency and risk of developing community-acquired pneumonia [30]. Early epidemiological research showed inverse associations between mean vitamin D status of 20 European countries and COVID-19 cases in those countries [31]. In a retrospective cohort study of 4,599 veterans with a positive SARS-CoV-2 test, after adjusting for all covariates, an inverse dose-response relationship between increasing vitamin D concentrations and decreasing probability of hospitalisation from COVID-19 was observed [32]. A number of meta-analyses supported a link between vitamin D deficiency and the severity of SARS-CoV-2 infection, with the largest indicating that those with low vitamin D levels were at an increased risk of ICU admission due to acute respiratory distress syndrome [18].

There is currently debate as to whether low serum vitamin D is caused by infection or if deficiency negatively affects immune defense. A meta-analysis of one population study and seven clinical studies that reported serum vitamin D levels pre-infection or on the day of hospital admission indicated that low serum levels are a predictor rather than a side effect of SARS-CoV-2 infection [33]. Furthermore, regression suggested a point of zero mortality at serum vitamin D levels ≥50 ng/mL [33].

Low-to-moderate evidence suggests possible benefits from vitamin D supplementation in adults and children with upper respiratory tract infections and influenza [34]. Similarly, there is mixed evidence for a beneficial role for vitamin D supplementation on need for ICU admission in patients with SARS-CoV-2 infection, with only two RCTs currently published [19,35]. Additional well-designed RCTs are needed to evaluate the efficacy of vitamin D supplementation in affecting SARS-CoV-2 infection outcomes. 

## 4. Adjunctive Therapy for Severe Disease

### 4.1. Vitamin C

Severe COVID-19 is characterised by the complications of pneumonia, acute respiratory distress syndrome (ARDS) and sepsis, typically requiring hospitalisation and intensive care for respiratory support [36]. Patients with pneumonia, ARDS and sepsis generally have severely depleted vitamin C levels [9]; comparable findings have been reported with COVID-19 patients [37]. Vitamin C supplementation of hospitalised patients with pneumonia has indicated decreased respiratory symptoms in the most severely ill and a dose-dependent decrease in the duration of hospital stay [29]. Patients with sepsis and ARDS in intensive care require gram doses of vitamin C to restore optimal vitamin C status; this is typically administered parenterally [9]. A recent RCT in septic patients with ARDS indicated decreased mortality and increased ICU and hospital free days in the group who received intravenous vitamin C (200 mg/kg/day) [38].

The World Health Organisation in 2020 highlighted intravenous vitamin C as a potential adjunctive therapy for patients with critical COVID-19 [39]. To date, five intervention trials have been published. The first trial to be carried out in patients with COVID-19-related pneumonia indicated a trend towards decreased 28-day mortality in the most severely ill patients who received intravenous vitamin C (24 g/day) [40]. Unfortunately, this trial was terminated early due to decreasing numbers of patients. Another trial that administered high-dose vitamin C (~28 g/d) to COVID-19 patients, who were receiving hydroxychloroquine, azithromycin, zinc, and vitamin D3, showed quicker recovery (symptom free and discharged from hospital) [41]. Three other trials in patients with severe COVID-19 who were administered lower doses of intravenous vitamin C (50 mg/kg/day and 6–8 g/day) showed no effects on mortality, but did find patients became symptom free earlier and spent fewer days in hospital [42], and had lower body temperature and improvements in oxygen saturation and respiratory rate [43,44]. Further trials are currently underway.

### 4.2. Vitamin D

Adjunct vitamin D supplementation in children with pneumonia did not reveal any significant reduction in the duration of hospitalisation, resolution of fever or acute illness, or mortality rate [45]. However, in adults with community-acquired pneumonia, vitamin D supplementation may benefit those with deficiency [46]. In the case of COVID-19, meta-analysis of two RCTs and a quasi-experimental study failed to show significant differences with vitamin D supplementation on various patient outcomes, including mechanical ventilation and mortality [19,47].

The potential for vitamin D to reduce inflammatory responses suggests it could function as an adjunctive therapy for recovery from COVID-19. A two-week 5000 IU oral vitamin D supplementation reduced the time to recovery for cough and loss-of-taste among patients with sub-optimal vitamin D status [48]. A study using 60,000 IU vitamin D for 8-10 days in individuals with low vitamin D levels showed a significant reduction in inflammatory markers as compared to standard care [49]. On the other hand, in a multicenter double-blind RCT, hospitalised patients (*n* = 120) receiving a single oral dose of 200,000 IU vitamin D did not show a significant reduction in hospital length of stay as compared to placebo [50]. Oral administration with calcifediol (25(OH)D) rather than vitamin D, raises serum 25(OH)D levels much more rapidly and may improve immune function and patient outcomes [51,52,53,54]. Further well designed RCTs are needed to determine the potential to use vitamin D in adjunctive therapy for COVID-19.

## 5. Attenuating Ongoing Sequelae

### 5.1. Vitamin C

The post-acute sequelae of COVID-19 (or ‘long COVID’) is characterised by persistent physical, cognitive and psychological symptoms which can significantly impair quality of life [55]. These include fatigue, muscle and joint pain, shortness of breath, chest pain, cough, and headache, as well as cognitive impairment, memory loss, anxiety and sleep disorders. Fatigue is a common symptom of viral infections in general and is one of the most frequently reported symptoms in long COVID [56]. Of note, fatigue, lethargy and low mood are early, preclinical symptoms of the vitamin C deficiency disease scurvy, whilst muscle and joint pain are common symptoms of clinical scurvy [57,58]. The vitamin C status of people with long COVID has not yet been assessed. Nevertheless, vitamin C intervention has been shown to improve symptoms of fatigue in people with various acute and chronic conditions, including herpes zoster infection [59], and has been proposed as a feasible therapy for the post viral fatigue of long COVID [60]. Furthermore, vitamin C supplementation has been reported to ameliorate pain in various acute and chronic conditions, including pain associated with viral infections [61].

Although not yet published, some current vitamin C and COVID trials (such as LOVIT-COVID) are assessing the longer-term health-related quality of life outcomes of short-term intravenous vitamin C administration to patients with COVID-19. However, it is unlikely that four days of intravenous vitamin C administration during critical COVID will have significant longer-term quality of life effects as cessation of vitamin C administration can result in a return to low baseline levels of vitamin C in many patients [38,62]. Thus, in order to see potential prevention of, or recovery from, long COVID ongoing daily vitamin C administration in appropriate dosages would be required. This remains to be established.

### 5.2. Vitamin D

Investigators are beginning to publish studies examining the relationship between vitamin D and long-term effects following SARS-CoV-2 infection and COVID-19. A recent study investigated the potential link between serum vitamin D levels and fatigue and reduced exercise tolerance in 149 patients at a median of 79 days after COVID-19 illness [63]. No relationship between vitamin D levels and ongoing ill-health was found after multivariable regression analysis. This relationship requires additional research and some vitamin D supplementation studies (e.g., VitD-COVID19) are examining physical activity as a secondary outcome.

## 6. Immunisation Support

### 6.1. Vitamin C

Vaccination against SARS-CoV-2 and the resultant activation of the immune system is likely to result in enhanced utilisation of immune supportive micronutrients, such as vitamin C. In cases of severe vitamin C deficiency, infants have been observed to go into shock following routine childhood vaccinations; rapid vitamin C administration was able to rescue many cases [64,65]. Animal studies have indicated that administration of vitamin C during vaccination or antigen challenge can stimulate an earlier and higher antibody response [66,67,68]. 

In 2019, the World Health Organization highlighted vaccine hesitancy as one of the top 10 threats to global health [69]. In the case of COVID-19 vaccination, a survey conducted in the USA found that 70% of vaccine-hesitant adults were worried about potential side effects of COVID-19 vaccines [70]. As discussed above, vitamin C can potentially attenuate symptoms of fatigue and pain [60,61]. Furthermore, decreased anaphylaxis and mortality from shock have been observed in vitamin C supplemented animals following challenge of passively sensitised animals [68]. As such, studies in people at risk of vitamin C deficiency who are receiving SARS-CoV-2 vaccines appear warranted.

### 6.2. Vitamin D

Vitamin D activates transcription of numerous genes involved in immune system support [3]. Research has suggested that low vitamin D status was associated with poorer hepatitis B vaccine response [71]. Meta-analysis of vitamin D deficiency and immunogenic response to influenza vaccine indicated lower seroprotection rates in response to vaccination against specific strains of influenza A and B virus in vitamin D deficient patients [72]. However, calcitriol co-administered intramuscularly with a commercially available influenza vaccine in 175 human volunteers provided no significant differences in hemagglutination titers at one or three months postvaccination [73]. In addition, initial research into vitamin D status and antibody response to COVID-19 mRNA vaccination in healthy adults did not show any significant association [74]. However, vitamin D may be able to support immune functions other than antibody response [75].

## 7. Summary and Conclusions

Immune support by micronutrients can be provided at various stages along the disease spectrum, from prevention of infection to adjunctive therapy for mild and severe disease, and its long-term sequelae, as well as support during immunisation. Table 1 summarises the evidence for the role of vitamins C and D in these different phases of immune support for SARS-CoV-2 and COVID-19, or general respiratory infections. Of note, there is significant overlap in the risk factors for vitamins C and D deficiency [16,76] and those for severe COVID-19 morbidity and mortality [23]. Therefore, restoring optimal vitamin C and D status in people with risk factors for both COVID-19 and vitamin deficiency may help to attenuate not only the risk of infection, but also the severity of complications.

**Figure 1:**
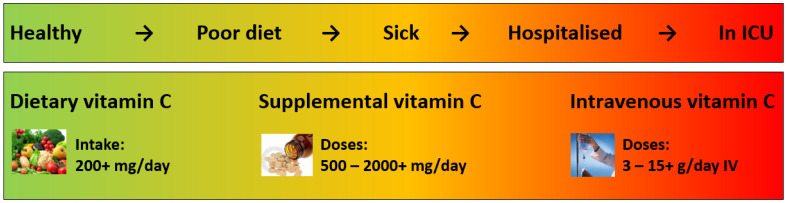
Vitamin C requirements along the spectrum of illness. Routs of administration and doses are those recommended or typically used. ICU, intensive care unit; IV, intravenous.

Vitamin C has numerous well-known immune supportive roles; however, its efficacy depends on appropriate timing, doses and rout of administration. Figure 1 summarises the different requirements for vitamin C along the spectrum of illness. To date, studies have indicated minimal effect on risk of SARS-CoV-2 and other upper respiratory tract infections with oral vitamin C, unless the person is under enhanced stress. However, there is some evidence that vitamin C can attenuate the duration and severity of respiratory and SARS-CoV-2 infections, thus potentially preventing progression to more severe conditions such as pneumonia, ARDS, sepsis and COVID-19. Intravenous vitamin C is typically administered to patients as adjunctive therapy once they enter into intensive care and preliminary studies indicate potential improvements in patients with ARDS and COVID-19. Larger trials are currently underway.

Vitamin D is implicated in numerous biological activities of the innate and adaptive immune system and observational studies suggest an inverse relationship between serum vitamin D concentrations and risk or severity of COVID-19. Some RCTs show vitamin D supplementation could reduce SARS-CoV-2 positivity, but data on ICU admission or all-cause mortality in those patients with moderate to severe disease is mixed. While further well-designed studies are needed, the evidence from prior work with respiratory tract infections and recent COVID-19 studies suggest taking a vitamin D supplement to reach serum vitamin D levels greater than 30 ng/mL is safe and could reduce risks associated with COVID-19.

To date, minimal research has been carried out around the potential effects of micronutrients on mitigating the debilitating symptoms of long COVID. Therefore, additional good-quality studies are needed in this area. Future studies investigating micronutrient support during immunisation should focus on people with comorbidities and other risk factors for both COVID-19 and vitamin deficiency. Furthermore, these studies should not focus exclusively on the antibody response, but also consider the other multiple levels of immune support offered by these vitamins, including potentially attenuating vaccine side effects. More research in these areas may provide evidence that could help with mitigating vaccine hesitancy.

## Figures and Tables

**Table 1:** Summary of multi-level immune support by vitamins C and D for SARS-CoV-2 infection and COVID-19, or general respiratory infections.

	Vitamin C		Vitamin D	
	SARS-CoV-2 and COVID-19*	General Respiratory Infections	SARS-CoV-2 and COVID-19	General Respiratory Infections
**Prevention of infection**	X risk reduction in case-control study [12]	X common cold risk in general population [11] ↓ common cold risk in people under stress [11]	↑ risk observed if low status [18]	↑ risk of acute RTI observed if low status [17] ↓ risk in people with low status if given vitD daily [20,21,22]
**Attenuating infection symptoms and severity**	? some evidence of decreased duration [25] ↑ rate of recovery [26]	↓ duration and severity of common cold [11] ↓ development of pneumonia [29]	↑ hospitalisation and ARDS observed if low status [18,32] X ICU admission (2 RCTs) [19]	↑ risk of pneumonia observed with deficiency [30] ? limited benefit in upper RTI and influenza [34]
**Adjunctive therapy for severe disease**	? some evidence of decreased mortality [40] ↑ rate of recovery [41,42] ↑ oxygenation [43,44]	↓ hospital stay in pneumonia [29] ↓ mortality, ICU and hospital stay in ARDS [38]	X mechanical ventilation or mortality (2 RCTs) [47]	X resolution or mortality in childhood pneumonia [43] ? may benefit those with deficiency [46]
**Attenuating ongoing sequelae**	? as yet unknown effects	↓ fatigue and pain in viral infections [60,61]	X fatigue, exercise tolerance [63]	? not assessed
**Immunisation support**	? as yet unknown effects	↑ antibody response in animals [66,67,68] ↓ anaphylaxis, shock mortality in animals [68] ↓ post immunisation shock in deficient infants [64,65]	X antibody response to mRNA vaccine in healthy adults [74]	↓ immunogenic response to some influenza vaccines if deficient [72] X hemagglutination titres following influenza vaccine [75]

* X no effect, ? uncertain effect, ↓ decreased, ↑ increased. Individual studies are cited where meta-analyses are not available. Some studies are observational only. ARDS; acute respiratory distress syndrome, RCTs; randomised controlled trials, RTI; respiratory tract infections.
